# Draft Genome Sequence of *Polaromonas glacialis* Strain R3-9, a Psychrotolerant Bacterium Isolated from Arctic Glacial Foreland

**DOI:** 10.1128/genomeA.00695-14

**Published:** 2014-07-10

**Authors:** Zhiyong Wang, Xulu Chang, Xu Yang, Li Pan, Jun Dai

**Affiliations:** a School of Bioscience and Bioengineering, South China University of Technology, Guangzhou, Guangdong, China; b BGI-Shenzhen, Shenzhen, Guangdong, China; c China Center for Type Culture Collection (CCTCC), College of Life Sciences, Wuhan University, Wuhan, Hubei, China; d Key Laboratory of Fermentation Engineering (Ministry of Education), Hubei Provincial Cooperative Innovation Center of Industrial Fermentation, College of Bioengineering, Hubei University of Technology, Wuhan, Hubei, China

## Abstract

Here we report the draft genome sequence of the psychrotolerant *Polaromonas glacialis* strain R3-9, isolated from Midtre Lovénbreen glacial foreland near Ny-Alesund, Svalbard Archipelago, Norway.

## GENOME ANNOUNCEMENT

The genus *Polaromonas*, a member of the family *Comamonadaceae*, phylum *Proteobacteria*, was first described by Irgens et al. (1), to accommodate Gram-negative, rod-shaped, psychrophilic bacteria. Since the discovery of the genus, seven validly described species have been isolated from various environments such as tap water, marine water, sediment, soil and glacier cryoconite: *Polaromonas vacuolata* (1), *Polaromonas naphthalenivorans* (2), *Polaromonas aquatic* (3), *Polaromonas hydrogenivorans* (4), and *Polaromonas jejuensis* (5). Darcy et al. (6) have reported that members of the genus *Polaromonas* are among the dominant bacteria of glacial ice and glacial sediments worldwide, including polar and high elevation environments. During a study of the bacterial diversity in the foreland of the high Arctic glacier Midtre Lovénbreen (N 78°53.704′ E 12°05.262′) in the Svalbard Archipelago, Norway, strain R3-9 (CCTCC AB 2014154) was isolated from a soil sample. A 16S rRNA gene analysis revealed that strain R3-9 shares high identity with the type strain of *Polaromonas glacialis* Cr4-12^T^. The 16S rRNA gene sequence similarity between strain R3-9 and its phylogenetically closely related neighbor was 98.54%, suggesting that strain R3-9 is a member of the *P. glacialis* species. Because the cells are able to grow well at relatively low temperatures, strain R3-9 is of interest for genomic research.

The genome of the strain was sequenced on the Illumina HiSeq2000 platform using a 90-bp paired-end library with an insert size of 500 bp. Following the manufacturer’s protocol, 142.69-fold coverage data and a total of 754 Mb of filtered paired-end reads were obtained. All reads were assembled into 59 contigs and 27 scaffolds using SOAPdenovo version 1.05 (7). Protein-coding sequences were predicted and analyzed using Glimmer 3.0 (8), and functional annotation of genes was performed by BLASTp searching of the KEGG (9), Clusters of Orthologous Groups (COG) (10), Swiss-Prot (11), TrEMBL (12), and NR (13) databases. rRNA and tRNA genes were detected by using RNAmmer (14) and tRNAscan-SE (15), respectively. Transposons were predicted by RepeatMasker and RepeatProteinMasker (16), and tandem repeat sequences were analyzed by Tandem Repeats Finder (17).

The information contained in the draft genome comprised 5,284,042 bp of sequence data, with a mean GC content of 62.27%, and the data cover a total of 5,375 predicted coding sequences (CDSs), 37 pseudogenes, 60 tRNAs, 3 rRNAs, 112 minisatellite DNAs, 47 microsatellite DNAs, and 4,567 proteins. The number of tandem repeat sequences was 198, and the total length of the tandem repeat sequences was 19,289 bp, which makes up 0.36% of the genome. Among the 4,567 genes identified, 4,113 were classified into 34 certain functional KEGG sets.

Knowing the genome sequence of *P. glacialis* strain R3-9 allows us to begin developing an integrated understanding of the physiology, genetics, ecology, and evolution of this psychrophilic bacterium.

### Nucleotide sequence accession number.

The genome sequence has been deposited at DDBJ/EMBL/GenBank under accession number JMDZ00000000. The version described in this paper is the first version.
